# Genetic Variation in the *NOC* Gene Is Associated with Body Mass Index in Chinese Subjects

**DOI:** 10.1371/journal.pone.0069622

**Published:** 2013-07-26

**Authors:** Yi-Cheng Chang, Yen-Feng Chiu, Pi-Hua Liu, Siow Wei Hee, Tien-Jyun Chang, Yi-Der Jiang, Wei-Jei Lee, Po-Chu Lee, Hui-Yi Kao, Juey-Jen Hwang, Lee-Ming Chuang

**Affiliations:** 1 Department of Internal Medicine, National Taiwan University Hospital, Taipei, Taiwan; 2 Genomics Research Center, Academia Sinica, Taipei, Taiwan; 3 Degree Program of Translational Medicine, National Taiwan University, Taipei, Taiwan; 4 Division of Biostatistics and Bioinformatics, Institute of Population Health Sciences, National Health Research Institutes, Miaoli, Taiwan; 5 Clinical Informatics and Medical Statistics Research Center, College of Medicine, Chang Gung University, Taoyuan, Taiwan; 6 Department of Surgery, Ming-Sheng General Hospital, Taoyuan, Taiwan; 7 Department of General Surgery, National Taiwan University Hospital, Taipei, Taiwan; 8 Graduate Institute of Clinical Medicine, College of Medicine, National Taiwan University, Taipei, Taiwan; McGill University, Canada

## Abstract

Circadian clock genes are critical regulators of energy homeostasis and metabolism. However, whether variation in the circadian genes is associated with metabolic phenotypes in humans remains to be explored. In this study, we systemically genotyped 20 tag single nucleotide polymorphisms (SNPs) in 8 candidate genes involved in circadian clock, including *CLOCK*, *BMAL1*(*ARNTL*), *PER1*, *PER2*, *CRY1*, *CRY2*, *CSNK1E,*, and *NOC*(*CCRN4L*) in 1,510 non-diabetic Chinese subjects in Taipei and Yunlin populations in Taiwan. Their associations with metabolic phenotypes were analyzed. We found that genetic variation in the *NOC* gene, rs9684900 was associated with body mass index (BMI) (*P* = 0.0016, Bonferroni corrected *P* = 0.032). Another variant, rs135764 in the *CSNK1E* gene was associated with fasting glucose (*P* = 0.0023, Bonferroni corrected *P* = 0.046). These associations were consistent in both Taipei and Yunlin populations. Significant epistatic and joint effects between SNPs on BMI and related phenotypes were observed. Furthermore, *NOC* mRNA levels in human abdominal adipose tissue were significantly increased in obese subjects compared to non-obese controls.

**Conclusion:**

Genetic variation in the *NOC* gene is associated with BMI in Chinese subjects.

## Introduction

The rotation of the earth around its axis generates the dark and light cycles, and organism living on earth developed circadian rhythms to adapt their activities to the dark and light cycles. In mammals, the central clock is located in the suprachiasmatic nucleus (SCN). The SCN clock is composed of single-cell neuronal oscillators that generate a coordinated output, which controls the systemic circadian rhythm. SCN neurons respond to external stimuli such as light or nutrients by adjusting their oscillatory activity [1]–[3]. In addition to external stimuli, the central circadian rhythm is also controlled by a intrinsic transcriptional auto-regulatory feedback loop. This feedback loop is composed of the transcriptional activators CLOCK and BMAL1 and their target genes *PERs* and *CRYs*. The *CLOCK* gene encodes a transcription factor, CLOCK, which dimerizes with BMAL1 to activate downstream genes, including *PERs* and *CRYs* [1]–[3]. PERs and CRYs oligomerize and translocate to the nucleus to inhibit CLOCK- BMAL1-mediated transactivation, thereby forming a negatively feedback loop. This autoregulatory loop is post-translationally regulated by the casein kinases, which target the PER proteins for degradation [1]–[3]. Another important circadian gene is the *NOC* gene, which encodes a deadenylase, nocturnin, that removes the 3 ′ adenosine residue from the mRNA transcripts of target genes at a post-transcriptional level to regulate gene function [4].

Several clinical studies have shown association between disturbed circadian rhythm and adverse metabolic consequence [5], [6]. Furthermore, mouse models with a targeted ablation of circadian genes also displayed abnormal energy metabolism. For example, *Clock* mutant mice developed hyperphagia, obesity, hyperlipidemia, and hyperglycemia [7]. *Per2* knockout mice showed an alteration in lipid metabolism, with a decrease in levels of triglyceride and free fatty acid [8]. *Nocturnin* knockout mice exhibited a resistance to diet-induced obesity, decreased triglycerides levels and improved insulin sensitivity [9]. These data indicate a critical role of circadian genes in energy homeostasis, glucose, and lipid metabolism.

In this present study, we systemically analyzed the relationship between variations in circadian genes and metabolic phenotypes in general populations in Taiwan. We found that genetic variation in the *NOC* gene was associated with body mass index (BMI). *NOC* expression in adipose tissue was increased in obese subjects. These data suggested a potential role for *NOC* in obesity.

## Results

**Figure 1:**
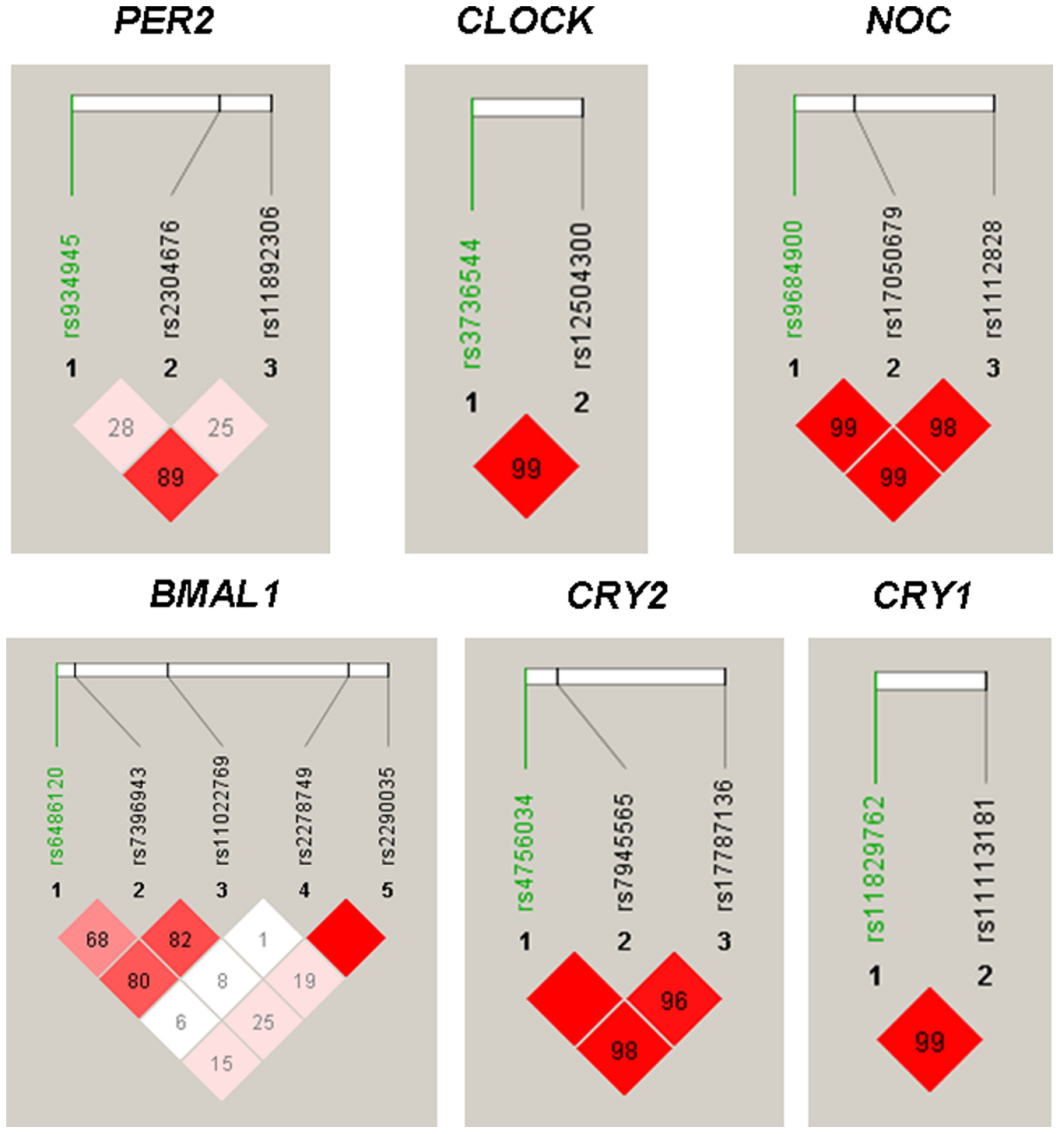
Graphical representation of linkage disequilibrium (LD) between SNPs. Pairwise LD coefficients D'×100 are shown in each cell (D' values of 1.0 were not shown). The standard color scheme of Haploview was applied for LD color display (LOD score ≧2 and D' = 1 in bright red; LOD score ≧2 and D'<1 in blue; LOD score <2 and D' = 1 in shade of pink; LOD score <2 and D'<1 in white).

The clinical and biochemical characteristics of study participants are summarized in Table 1. The nucleotide composition, chromosomal position, and minor allele frequencies for all genotyped SNP are summarized in Table 2. On average, the genotype call rate was 97.6%. All SNP were in Hardy-Weinberg equilibrium. The LD patterns between SNPs are shown in Figure 1.

**Table 1:** Characteristics of study participants.

	Taipei participants	Yunlin participants	Total
Number	760	750	1,510
Age	64.52 (13.64)	47.51 (12.39)	56.06 (15.56)
Male sex (%)	56.6 (%)	44.53 (%)	50.06%
Obesity (%)	12.8 (%)	26.28 (%)	19.4%
Body mass index (kg/m2)	23.64 (3.14)	24.89 (3.89)	24.25 (3.58)
Systolic blood pressure (mmHg)	127.62 (16.98)	128.25 (16.93)	127.9 (16.95)
Diastolic blood pressure (mmHg)	76.15 (10.1)	84.25 (10.94)	80.1 (11.27)
Fasting serum triglyceride (mg/dL)	112.3 (63.83)	119.58 (90.24)	115.9 (78.14)
Fasting plasma glucose (mg/dL)	96.84 (13.11)	90.93 (20.26)	93.90 (17.28)

Data are presented as mean (S.D.) or percentage.

**Table 2:** SNP information.

Gene	SNP name	Chr.	Position(kb)	Major/minor Allele	Minor allele frequency	HW *P*-value
*PER2*	rs2304676	2	238844643	C/T	0.23	0.71
*PER2*	rs11892306	2	238853208	G/T	0.32	1
*CLOCK*	rs3736544	4	56004739	G/A	0.35	0.74
*CLOCK*	rs12504300	4	56043274	C/G	0.41	0.53
*NOC*	rs9684900	4	140160847	G/A	0.27	0.48
*NOC*	rs17050679	4	140165667	G/C	0.43	0.27
*NOC*	rs1112828	4	140176410	T/G	0.4	0.35
*BMAL1*	rs6486120	11	13280708	T/G	0.48	0.76
*BMAL1*	rs7396943	11	13285545	C/G	0.4	0.28
*BMAL1*	rs11022769	11	13308971	A/C	0.45	0.68
*BMAL1*	rs2278749	11	13354444	G/A	0.14	0.45
*BMAL1*	rs2290035	11	13364337	T/A	0.25	0.94
*CRY2*	rs4756034	11	45832521	A/G	0.39	0.6
*CRY2*	rs7945565	11	45835558	A/G	0.26	0.43
*CRY2*	rs17787136	11	45851202	C/G	0.12	0.8
*CRY1*	rs11829762	12	105912668	C/A	0.22	0.83
*CRY1*	rs11113181	12	105992381	A/G	0.3	0.95
*PER1*	rs2304911	17	7991694	T/C	0.22	0.32
*CSNK1E*	rs135764	22	37040348	G/A	0.16	0.85

HW *P*-values, empirical *P*-values of the χ^2^ test for Hardy-Weinberg equilibrium.

### SNP association with metabolic phenotypes

Single-locus SNP associations with metabolic phenotypes are summarized in Table 3. Among the 20 genotyped SNPs, the A allele at rs9684900 in the *NOC* gene was associated with higher BMI (*P* = 0.0016, Bonferroni adjusted *P* = 0.032) (Table 4). The estimated effect size associated with each A allele was 0.46 kg/m^2^. This direction associations are consistent in both Taipei and Yunlin populations (*P* = 0.0045 and *P* = 0.091, respectively)(Table S1). We also observed nominal associations of rs17050679 in the *NOC* gene with BMI (*P* = 0.0057, Bonferroni corrected *P* = 0.11) and fasting plasma triglycerides (*P* = 0.0037, Bonferroni corrected *P* = 0.074). The directions of association with BMI (*P* = 0.021 in Taipei and *P* = 0.074 in Yunlin population) and fasting plasma triglycerides (*P* = 0.026 in Taipei and *P* = 0.046 in Yunlin population) are also consistent in both study populations (Table S1).

**Table 3:** SNP association with metabolic phenotypes.

		BMI		SBP		DBP		Fasting TG		Fasting glucose	
SNP	Gene	Estimate	*P*	Estimate	*P*	Estimate	*P*	Estimate	*P*	Estimate	*P*
rs934945	*PER2*	−0.004	0.52	0.0069	0.19	0.0007	0.91	−0.025	0.26	0.00244	0.65
rs2304676	*PER2*	0.0056	0.38	0.012	**0.036**	0.0057	0.35	0.02	0.39	0.016	**0.0043**
rs11892306	*PER2*	−0.0013	0.82	0.00066	0.9	0.0064	0.25	0.0081	0.7	0.0095	0.069
rs3736544	*CLOCK*	−0.002	0.76	−0.0036	0.52	0.0037	0.53	0.011	0.66	0.0086	0.16
rs12504300	*CLOCK*	−0.0032	0.56	−0.007	0.14	−0.0022	0.67	−0.0019	0.92	0.0085	0.079
rs9684900	*NOC*	0.019	**0.0016**	0.001	0.85	−0.00054	0.93	0.042	0.055	0.0047	0.38
rs17050679	*NOC*	−0.015	**0.0057**	−0.0057	0.22	−0.0074	0.15	−0.056	**0.0037**	0.0032	0.5
rs1112828	*NOC*	0.0045	0.4	−0.0027	0.56	−0.0061	0.24	0.043	**0.029**	−0.0027	0.58
rs6486120	*BMAL1*	0.005	0.35	0.006	0.2	0.0056	0.27	0.016	0.41	−0.00069	0.89
rs7396943	*BMAL1*	0.011	**0.047**	0.008	0.093	0.0082	0.12	0.0074	0.71	−0.0005	0.92
rs11022769	*BMAL1*	−0.0005	0.92	−0.00703	0.13	−0.0062	0.24	0.0049	0.81	0.0019	0.69
rs2278749	*BMAL1*	0.01	0.17	0.0054	0.42	0.0022	0.76	0.052	0.067	0.0019	0.78
rs2290035	*BMAL1*	−0.0024	0.7	0.0049	0.36	0.003	0.62	0.023	0.31	0.0016	0.78
rs4756034	*CRY2*	−0.0038	0.56	0.0046	0.41	0.0088	0.13	0.022	0.35	0.0045	0.46
rs7945565	*CRY2*	−0.0076	0.2	0.0034	0.51	0.0015	0.8	−0.002	0.93	0.0045	0.38
rs17787136	*CRY2*	0.0043	0.61	−0.0012	0.87	0.0096	0.24	0.0347	0.26	−0.0077	0.3
rs11829762	*CRY1*	−0.01	**0.036**	−0.007	0.21	−0.0085	0.16	−0.0185	0.43	−0.0035	0.54
rs11113181	*CRY1*	0.014	**0.014**	0.0069	0.17	0.007	0.21	0.0022	0.92	0.0083	0.11
rs2304911	*PER1*	−0.0043	0.5	0.0119	**0.031**	0.013	**0.031**	0.017	0.47	0.0066	0.23
rs135764	*CSNK1E*	0.01	0.18	0.004	0.53	0.0057	0.42	−0.013	0.64	0.019	**0.0023**

BMI: body mass index, SBP: systolic blood pressure, DBP: diastolic blood pressure, TG: triglycerides; Bold indicates *P*<0.05. Data are presented as mean (S.D.).All of these traits were log-transformed in the regression analysis.

**Table 4:** Metabolic phenotypes according to *NOC* rs9684900 genotypes.

Phenotypes	GG	GA	AA	*P*
BMI (kg/m2)	24.01 (3.55)	24.48 (3.59)	24.93 (3.63)	**0.0016**
SBP (mmHg)	128.06 (16.96)	127.45 (17)	129.92 (16.55)	0.85
DBP (mmHg)	80.24 (11.28)	79.95 (11.21)	80.81 (12.17)	0.93
Fasting TG (mg/dL)	113.02 (72.27)	118.92 (86.93)	122.4 (70.32)	0.055
Fasting glucose (mg/dL)	93.58 (14.63)	94.09 (20.64)	95.24 (15.48)	0.38

BMI: body mass index, SBP: systolic blood pressure, DBP: diastolic blood pressure, TG: triglycerides; Bold indicates *P*<0.05. Data are presented as mean (S.D.).

Another variant, the A allele at rs135764 in the *CSNK1E* gene was significantly associated with fasting plasma glucose (*P* = 0.0023, Bonferroni corrected *P* = 0.046) (Table 5) with concordant directions of association in Taipei and Yunlin populations (*P* = 0.058 and *P* = 0.021, respectively) (Table S1).

**Table 5:** Metabolic phenotypes according to *CSNK1E* rs135764 genotypes.

Phenotypes	GG	GA	AA	*P*
BMI (kg/m^2^)	24.16 (3.47)	24.49 (3.87)	24.54 (3.59)	0.18
SBP (mmHg)	127.81 (17.02)	128.17 (16.84)	127.45 (16.08)	0.53
DBP (mmHg)	80.01 (11.5)	80.39 (10.63)	81.47 (12.08)	0.42
Fasting TG (mg/dL)	116.02 (70.52)	113.59 (92.59)	133.63 (114.95)	0.64
Fasting glucose (mg/dL)	93.25 (15.17)	94.22 (13.93)	99.66 (29.7)	**0.** **0023**

BMI: body mass index, SBP: systolic blood pressure, DBP: diastolic blood pressure, TG: triglycerides; Bold indicates *P*<0.05. Data are presented as mean (S.D.).

### The effect of multi-locus interaction between SNPs on metabolic phenotypes

We next evaluated the effect of interaction between SNPs on metabolic phenotypes. Results of the multilocus interaction analyses using GMDR are summarized in Table 6. The most significant interactions with a good cross-validation consistency (CVC) were found between *NOC* rs17050679 and *NOC* rs9684900 (*P*<0.001, CVC: 8/10) for BMI and between *NOC* rs9684900 and *BMAL1* rs2290035 (*P* = 0.001, CVC: 7/10) for fasting glucose level.

**Table 6:** Multi-locus interaction on metabolic phenotypes.

Traits	# Loci	Polymorphism in model	Cross-validation consistency	*P* *
Body mass index	2	*NOC* rs17050679, *NOC* rs9684900	8/10	**<0.001**
Diastolic blood pressure	3	*PER2* rs11892306, *PER1* rs2304911, *CK1* rs2075983	5/10	**0.003**
Fasting triglycerides	2	*BMAL1* rs2278749, *CRY2* rs7945565	5/10	**0.012**
Fasting glucose	2	*NOC* rs9684900, *BMAL1* rs2290035	7/10	**0.001**

* permutated for 1,000 times and adjusted for age and sex.

### Joint effects of risk alleles on metabolic phenotypes

**Figure 2:**
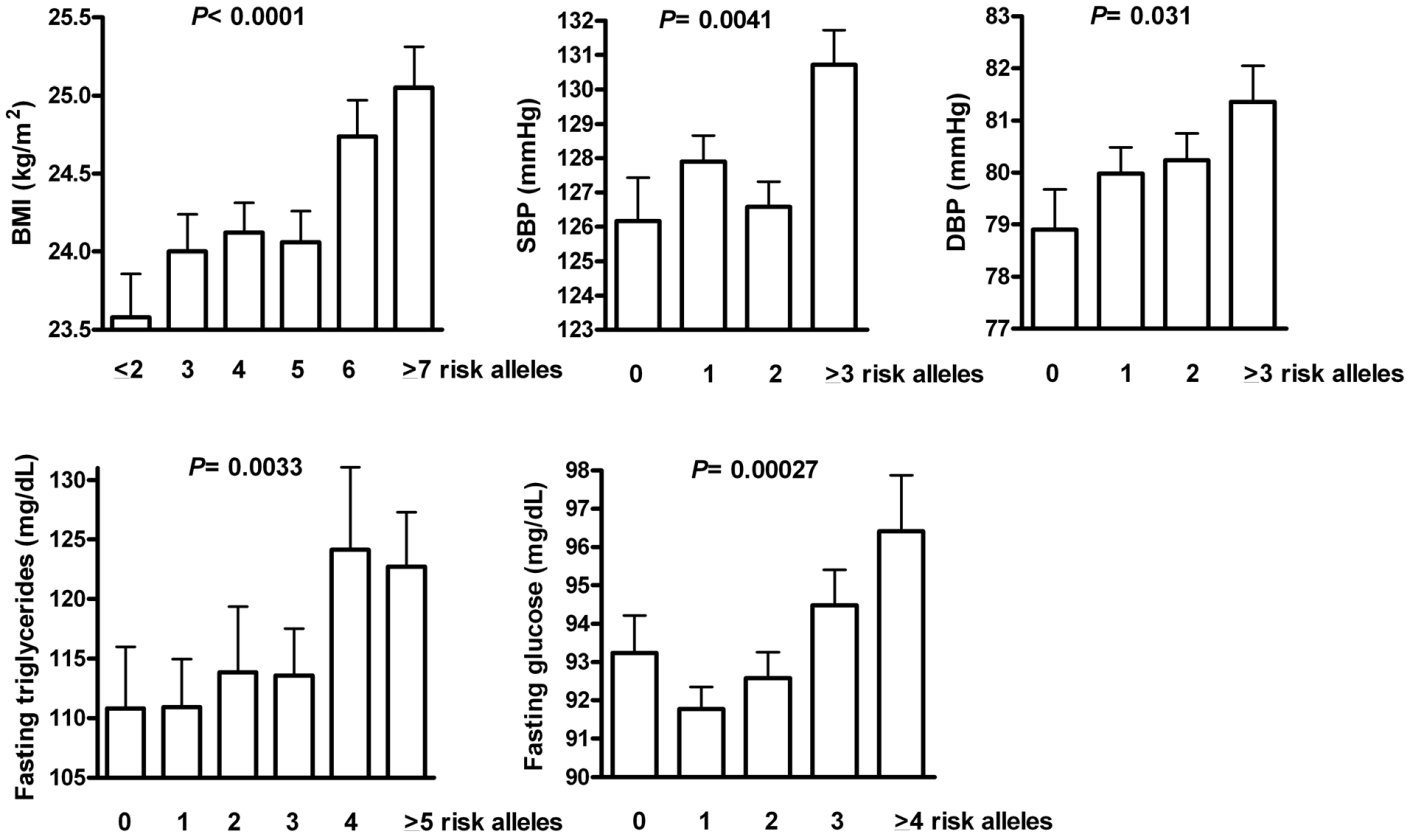
Distribution of body mass index (BMI), systolic (SBP) and diastolic blood pressure (DBP), fasting triglycerides, and fasting plasma glucose according to number of risk alleles.

We further evaluated the joint effects of risk alleles with nominal association (*P*<0.1) with metabolic phenotypes. The distribution of BMI, blood pressures, fasting triglycerides, and fasting glucose according to different number of risk alleles is shown in Figure 2. Increasing risk alleles were significantly associated with higher BMI (*P*<0.0001), systolic blood pressure (SBP) (*P* = 0.0041), DBP (*P* = 0.031), fasting triglycerides (*P* = 0.0033) and fasting glucose levels (*P* = 0.00027).

### *NOC* expression in adipose tissue between obese and non-obese subjects

**Figure 3:**
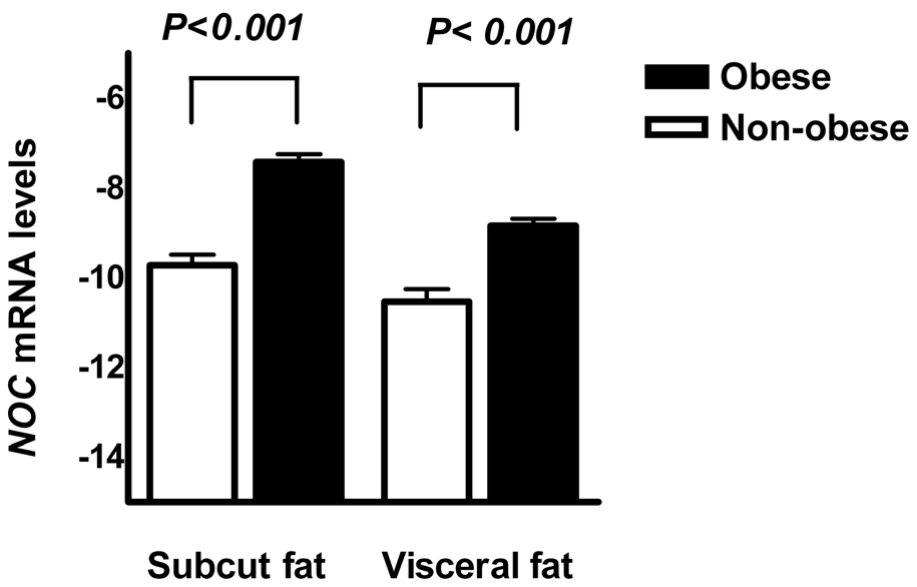
*NOC* expression in abdominal subcutaneous and visceral fat tissue between obese and non-obese subjects.

Nocturnin was previously reported to be a regulator of adipogenesis highly expressed in adipocytes [10]. We next explored whether *NOC* expression in adipose tissue was associated with obesity. We found that *NOC* expression was higher in obese subjects in either subcutaneous or visceral adipose tissue (*P*<0.001) (Figure 3).

## Discussion

In this present study, we systemically analyzed the association of tag SNPs in 8 circadian genes with metabolic phenotypes in 1,510 otherwise healthy Taiwanese individuals. We found that variation in the *NOC* gene was associated with BMI. Interestingly, *NOC* expression in abdominal adipose tissue was positively associated with obesity. Consistent with our findings, nocturnin has been shown to regulate adipogenesis by stimulating nuclear translocation of PPARγ [11] or by modulating mitotic clonal expansion during early adipogenesis [10]. Importantly, *Nocturnin* knockout mice were protected from diet-induced obesity [9]. These findings indicate nocturnin is an important regulator of adiposity. The molecular mechanism by which an intronic variant in the *NOC* gene influences phenotype is currently not known. It is most likely that the functional causative variant is in LD with this associated variant.

We also found a significant association between *CSNK1E* genetic variant and fasting glucose. The *CSNK1E* encodes for the casein kinase 1 epsilon, a serine/threonine kinase that phosphorylates PER proteins in the cytoplasm and triggers their proteasome-dependent degradation. In yeasts, glucose-dependent activation of casein kinase I catalyzes phosphorylation of Mth1, a glucose transporter gene (*HXT*) repressor, triggering degradation of Mth1 by the proteasome and leads to de-repression of *HXT* gene expression [12], [13]. These data suggest a potential role of casein kinase in glucose homeostasis.

Interestingly, we also found that an interaction between *NOC* and *BMAL1* genetic variants may affect fasting glucose levels. Loss of Bmal1 in mice has been shown to cause hyperglycemia due to impairment in β-cells function while deletion of *Noc* in mice causes higher fasting glucose. Although no main effect of *BMAL1* and *NOC* genetic variants on fasting glucose was observed, fasting glucose were significantly elevated in the presence of both genetic variants. These data indicates that a clinically evident phenotype may require a close interaction between 2 or more genetic factors.

However, our results were not consistent with a previous genome-wide association analysis reporting an association between *CRY2* variant and fasting glucose [14]. In our study, all tag SNPs in the *CRY2* gene were not associated with fasting plasma glucose. Our results are also not consistent with previous candidate-gene association studies reporting significant associations between *CLOCK* genetic variation and obesity [15]–[17]/metabolic syndrome [18], *PER2* genetic variation and obesity [19]/fasting plasma glucose [20], and *BMAL1* genetic variation and type 2 diabetes/hypertension [21]. Nevertheless, since we did not genotype the same SNPs (or tagSNPs) with previous studies, a direct comparison between studies is not possible. Heterogeneity between different ethnic groups may also confound the association.

The major limitation of this study is the lack of replication. However, the directions of association between *NOC* variant and BMI and between *CSNK1E* variant and fasting glucose are the same in both Taipei and Yunlin populations, indicating consistency in different populations. Further study is still warranted to confirm the association.

In conclusion, we systemically analyzed the association of tag SNPs in circadian genes with metabolic phenotypes. We identified associations of *NOC* genetic variation with BMI. A strong correlation of *NOC* expression in human adipose tissue with obesity was also observed. These data, together with previous animal models, suggest a substantial role of nocturnin in obesity.

## Materials and Methods

### Subjects

The studied population comprised 760 subjects recruited from the Health Management Center of National Taiwan University Hospital (NTUH) in Taipei and 750 subjects recruited from a community-based screening program in the Yunlin County in southern Taiwan. The detailed description of study subjects have been described previously [22], [23]. Patients with a history of diabetes or other major systemic diseases were excluded [22], [23]. Blood pressure was measured by mercury sphygmomanometer to the nearest 2 mmHg. Trained nurses took three separate readings at 1-minute intervals. Fasting glucose and triglycerides levels were measured by an automatic analyzer (Toshiba TBA 120FR, Toshiba Medical System Co., Japan). The study was approved by the National Taiwan University Hospital Research Ethics Committee. Written informed consent was obtained from each participant.

### Subjects for measurement of gene expression in adipose tissue

We recruited 60 non-diabetic subjects undergoing bariatric surgery or elective abdominal surgery such as cholecystectomy or partial hepatectomy at the Ming-Sheng General Hospital and the Yunlin branch of NTUH in Taiwan. Abdominal subcutaneous adipose tissues were sampled from patients in a fasting state prior to surgery and were immediately placed in liquid nitrogen until processing. The study was approved by the institutional review board of Ming-Sheng General Hospital and NTUH. Written informed consent was obtained from each patient.

### SNP genotyping

The Tagger program implemented in Haploview version 4.0 software (http://www.broad.mit.edu/mpg/haploview/) [24] was used to select tag SNPs (with a minor allele frequency threshold of 0.1) from the HapMap Chinese Beijing (CHB) databank (Rel 24/phase II). In total, 20 tag SNPs were selected to capture the genetic variation of 8 genes involved in circadian clock, including *CLOCK* (clock circadian regulator, Gene ID: 9575), *BMAL1* (*ARNTL*, aryl hydrocarbon receptor nuclear translocator-like, Gene ID: 406) *PER1* (period circadian clock 1, Gene ID: 5187), *PER2* (period circadian clock 2, Gene ID: 8864), *CRY1* (cryptochrome 1, Gene ID: 1407), *CRY2* (cryptochrome 2, Gene ID: 1408), *CSNK1E* (casein kinase 1, epsilon, Gene ID: 1454), and *NOC* (*CCRN4L*, CCR4 carbon catabolite repression 4-like, Gene ID: 25819). These 20 tag SNPs capture average 71.4% of all SNPs in these 8 genes with a minor allele frequency greater than 0.1 at *r* ^2^ of 0.7. Genotyping was performed using the GenomeLab SNPstream genotyping platform (Beckman Coulter, Brea CA, USA) and its accompanying SNPstream software suite. The concordance rate of genotyping based on this platform was 99.62%. The raw genotype and phenotype data were deposited as Data S1.

### Reverse transcription and quantitative real-time PCR

Total RNA was isolated using REzol^™^C&T Reagent (Protech, Taipei, Taiwan) and reverse transcribed with Superscript III kit (Invitrogen, Carlsbad, CA, USA) according to the manufacturer's instructions. PCR amplification was performed using LightCycler FastStart DNA master Plus SYBR (Roche, Mannheim, Germany). Each sample was analyzed in duplicate and the gene expression was normalized to that of the *PPIA* (cyclophilin A), a housekeeping gene. The primers used for *NOC* were PPH23949A (SA BioScience, Frederick, MD, USA). The primers used for *PPIA* (cyclophilin A) were forward: 5′-GCATACGGGTCCTGGCATCTTGTCC-3′ and reverse: 5′-ATGGTGATCTTCTTGCTGGTCTTGC-3′, respectively. The correlation (*R* ^2^) between Ct value and log cDNA input was 0.99 for *NOC* primers and 0.99 for *PPIA* primers (Figure S1A&S1B). The efficiency of primers for *NOC* and *PPIA* are 0.99 and 0.93, respectively. The slope between delta Ct (Ct of *PPIA* minus Ct of *NOC*) and log cDNA input was 17.1% (Figure S1C). There is no difference of Ct of *PPIA* between obese and non-obese subjects in either subcutaneous or visceral fat (Figure S1D).

### Statistical analyses

Data that were not normally distributed were logarithmically transformed to approximate normal distribution. A Hardy-Weinberg equilibrium (HWE) test was performed for each sequence variant before marker-trait association analysis was estimated by using the Haploview software. Linear regression with an additive genetic model was used to analyze SNP association with quantitative traits. Inter-marker linkage disequilibrium (LD) was measured by pairwise D' and *r* ^2^. Multi-locus interaction between SNPs on metabolic phenotypes was analyzed using the Generalized Multifactor Dimensionality Reduction (GMDR) computing package (http://www.ssg.uab.edu/gmdr/) with adjustment for age and sex. GMDR is a model-free combinatorial approach for detecting multi-locus interactions [25]. The combinations of all factors were classified into high- or low-risk groups based on the score-based statistic. The best n-factor model that yielded a minimum misclassification error was chosen (*n* = 1, 2, 3 in this study). The significance of the best n-factor model was assessed based on 1,000 permutations. To test for cumulative effects of genetic variants on the individual phenotypes, a subset of SNPs that had nominal associations (*P*<0.1) with metabolic phenotypes were collected as a risk-SNP set. Consequently, an individual can carry the number of risk alleles ranging from 0 to 2 times of the total number of risk SNPs. For instance, one individual can carry 0 up to 10 risk alleles for BMI, 0 up to 6 risk alleles for SBP or DBP, 0 up to 8 risk alleles for fasting serum triglycerides, and 0 up to 8 risk alleles for fasting plasma glucose. Subjects were then divided into 4, 5 or 6 groups based on the number of risk alleles for each phenotype. An ordinal variable coded as 1–4 for the 4 groups (or 1–5 for the 5 groups; 1–6 for 6 groups) was used as a covariate for testing the trend effect from the cumulative risk alleles on the individual phenotype using a linear regression with adjustment for age and sex.

## Supporting Information

Figure S1 Correlation between Ct value and log (cDNA input) for (A) *NOC* primers and (B) *PPIA* primers. (C) slope (dotted line) between delta Ct (Ct of *PPIA* minus Ct of *NOC*) and log (cDNA input) (D) Ct of *PPIA* (internal control) in subcutaneous and visceral fat in obese and non-obese subjects.
(TIF)

Table S1 SNP association with metabolic phenotypes according to study populations.
(DOC)

Data S1 (XLSX)
