# Supplementary figures and images for: Genetic Variation in the NOC Gene Is Associated with Body Mass Index in Chinese Subjects
Source: PLoS One. 2013 Jul 26;8(7):e69622. doi: 10.1371/journal.pone.0069622 (PMC3724939; doi:10.1371/journal.pone.0069622)

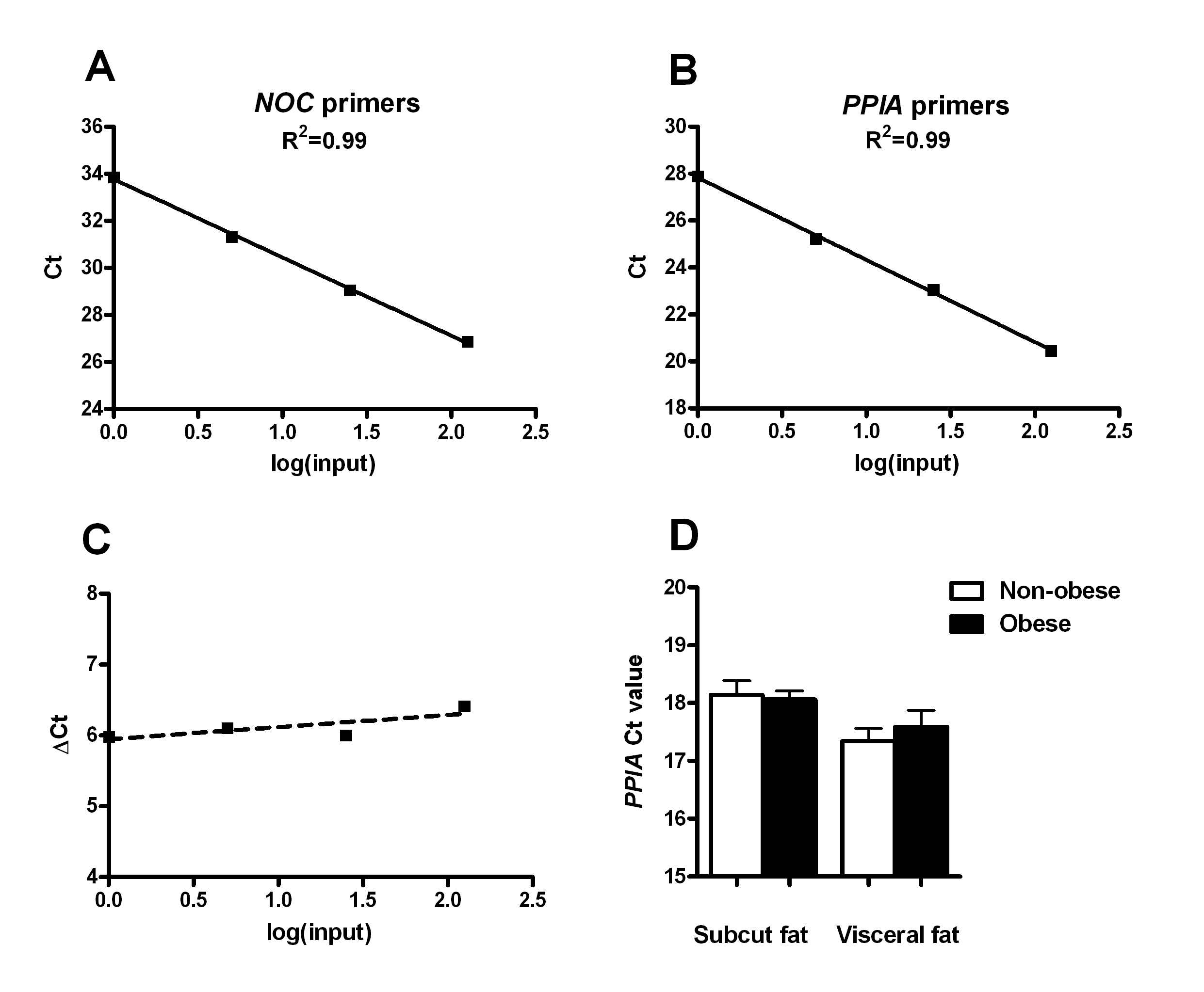

Supplement: Figure S1 — Correlation between Ct value and log (cDNA input) for (A) NOC primers and (B) PPIA primers. (C) slope (dotted line) between delta Ct (Ct of PPIA minus Ct of NOC) and log (cDNA input) (D) Ct of PPIA (internal control) in subcutaneous and visceral fat in obese and non-obese subjects. (TIF) [file pone.0069622.s001.tif]
