# Supplementary material for: Genetic Variation in the NOC Gene Is Associated with Body Mass Index in Chinese Subjects
Source: PLoS One. 2013 Jul 26;8(7):e69622. doi: 10.1371/journal.pone.0069622 (PMC3724939; doi:10.1371/journal.pone.0069622)
Supplement: Table S1 — SNP association with metabolic phenotypes according to study populations. (DOC) [file pone.0069622.s002.doc]

**Table S1.** SNP association with metabolic phenotypes according to study populations

|  |  | **BMI** |  |  |  | **SBP** |  |  |  | **DBP** |  |  |  |
| --- | --- | --- | --- | --- | --- | --- | --- | --- | --- | --- | --- | --- | --- |
|  |  | **Taipei** |  | **Yunlin** |  | **Taipei** |  | **Yunlin** |  | **Taipei** |  | **Yunlin** |  |
| **SNP** | **Gene** | **Estimate** | ***P*** | **Estimate** | ***P*** | **Estimate** | ***P*** | **Estimate** | ***P*** | **Estimate** | ***P*** | **Estimate** | ***P*** |
| rs934945 | *PER2* | -0.006 | 0.45 | -0.0017 | 0.86 | 0.0051 | 0.48 | 0.0093 | 0.21 | -0.00079 | 0.92 | 0.00054 | 0.94 |
| rs2304676 | *PER2* | 0.00069 | 0.93 | 0.013 | 0.17 | 0.015 | **0.041** | 0.01 | 0.18 | 0.0019 | 0.82 | 0.012 | 0.12 |
| rs11892306 | *PER2* | -0.014 | 0.079 | 0.0063 | 0.45 | -0.0058 | 0.4 | 0.0017 | 0.8 | 0.0011 | 0.89 | 0.0071 | 0.31 |
| rs3736544 | *CLOCK* | -0.0087 | 0.42 | 0.00097 | 0.9 | 0.0028 | 0.79 | -0.0063 | 0.34 | 0.017 | 0.094 | -0.0028 | 0.68 |
| rs12504300 | *CLOCK* | -0.0075 | 0.29 | 0.002 | 0.8 | -0.0074 | 0.26 | -0.0055 | 0.4 | 0.0012 | 0.87 | -0.0055 | 0.42 |
| rs9684900 | *NOC* | 0.022 | **0.0045** | 0.015 | 0.091 | 0.0031 | 0.66 | -0.0019 | 0.8 | 0.002 | 0.79 | -0.004 | 0.6 |
| rs17050679 | *NOC* | -0.016 | **0.021** | -0.014 | 0.074 | -0.01 | 0.11 | -0.0021 | 0.74 | -0.0066 | 0.34 | -0.0095 | 0.14 |
| rs1112828 | *NOC* | 0.0049 | 0.47 | 0.0063 | 0.44 | 0.00071 | 0.91 | -0.0039 | 0.55 | -0.0037 | 0.59 | -0.002 | 0.77 |
| rs6486120 | *BMAL1* | 0.000071 | 0.99 | 0.0064 | 0.42 | 0.000083 | 0.99 | 0.0078 | 0.23 | -0.0038 | 0.58 | 0.0084 | 0.21 |
| rs7396943 | *BMAL1* | -0.0029 | 0.68 | 0.023 | **0.0052** | 0.0014 | 0.83 | 0.012 | 0.085 | -0.0038 | 0.59 | 0.017 | **0.016** |
| rs11022769 | *BMAL1* | -0.00058 | 0.93 | 0.0027 | 0.74 | -0.0068 | 0.28 | -0.0035 | 0.59 | -0.0012 | 0.87 | -0.0044 | 0.52 |
| rs2278749 | *BMAL1* | 0.023 | **0.021** | -0.0015 | 0.89 | 0.0053 | 0.55 | 0.0061 | 0.51 | 0.0057 | 0.57 | 0.005 | 0.6 |
| rs2290035 | *BMAL1* | -0.0036 | 0.64 | -0.00091 | 0.92 | 0.011 | 0.12 | -0.0017 | 0.82 | -0.00059 | 0.94 | 0.0073 | 0.36 |
| rs4756034 | *CRY2* | 0.0011 | 0.91 | -0.007 | 0.39 | -0.0034 | 0.73 | 0.008 | 0.23 | 0.013 | 0.19 | 0.0059 | 0.39 |
| rs7945565 | *CRY2* | -0.011 | 0.15 | -0.0016 | 0.86 | 0.004 | 0.56 | 0.0059 | 0.42 | 0.0077 | 0.31 | 0.001 | 0.89 |
| rs17787136 | *CRY2* | 0.017 | 0.13 | -0.014 | 0.26 | -0.013 | 0.22 | 0.00058 | 0.95 | -0.014 | 0.23 | 0.016 | 0.11 |
| rs11829762 | *CRY1* | -0.0074 | 0.34 | -0.018 | 0.071 | 0.00088 | 0.9 | -0.014 | 0.099 | 0.002 | 0.8 | -0.014 | 0.091 |
| rs11113181 | *CRY1* | 0.01 | 0.18 | 0.017 | **0.048** | 0.0017 | 0.81 | 0.011 | 0.12 | 0.0031 | 0.68 | 0.005 | 0.48 |
| rs2304911 | *PER1* | -0.013 | 0.12 | 0.00051 | 0.96 | 0.0026 | 0.74 | 0.016 | **0.032** | -0.00083 | 0.92 | 0.019 | **0.014** |
| rs135764 | *CSNK1D* | -0.0029 | 0.76 | 0.023 | **0.031** | 0.0077 | 0.37 | 0.00046 | 0.96 | 0.016 | 0.096 | -0.0039 | 0.66 |

|  |  | **TG** |  |  |  | **Fasting glucose** |  |  |  |
| --- | --- | --- | --- | --- | --- | --- | --- | --- | --- |
|  |  | **Taipei** |  | **Yunlin** |  | **Taipei** |  | **Yunlin** |  |
| **SNP** | **Gene** | **Estimate** | ***P*** | **Estimate** | ***P*** | **Estimate** | ***P*** | **Estimate** | ***P*** |
| rs934945 | *PER2* | -0.0068 | 0.82 | -0.041 | 0.21 | -0.00091 | 0.89 | 0.0072 | 0.41 |
| rs2304676 | *PER2* | 0.0068 | 0.83 | 0.04 | 0.23 | 0.014 | **0.046** | 0.017 | **0.037** |
| rs11892306 | *PER2* | 0.01 | 0.73 | 0.0024 | 0.94 | -0.0013 | 0.84 | 0.018 | **0.023** |
| rs3736544 | *CLOCK* | 0.025 | 0.52 | 0.0088 | 0.76 | 0.01 | 0.28 | 0.0083 | 0.28 |
| rs12504300 | *CLOCK* | 0.0066 | 0.8 | -0.003 | 0.92 | 0.0035 | 0.56 | 0.014 | 0.065 |
| rs9684900 | *NOC* | 0.055 | 0.058 | 0.032 | 0.33 | 0.00066 | 0.92 | 0.0088 | 0.31 |
| rs17050679 | *NOC* | -0.058 | **0.026** | -0.055 | **0.046** | 0.003 | 0.61 | 0.0037 | 0.62 |
| rs1112828 | *NOC* | 0.047 | 0.069 | 0.038 | 0.19 | -0.003 | 0.6 | -0.0041 | 0.6 |
| rs6486120 | *BMAL1* | 0.0029 | 0.91 | 0.025 | 0.38 | 0.0029 | 0.62 | -0.0038 | 0.62 |
| rs7396943 | *BMAL1* | 0.014 | 0.6 | -0.0038 | 0.9 | -0.00034 | 0.95 | -0.00092 | 0.91 |
| rs11022769 | *BMAL1* | -0.001 | 0.97 | 0.018 | 0.54 | 0.005 | 0.39 | -0.0026 | 0.74 |
| rs2278749 | *BMAL1* | 0.049 | 0.19 | 0.054 | 0.19 | 0.0055 | 0.51 | -0.0059 | 0.56 |
| rs2290035 | *BMAL1* | 0.021 | 0.48 | 0.026 | 0.45 | -0.0018 | 0.79 | 0.0056 | 0.53 |
| rs4756034 | *CRY2* | 0.011 | 0.76 | 0.021 | 0.48 | -0.007 | 0.44 | 0.011 | 0.17 |
| rs7945565 | *CRY2* | 0.0053 | 0.85 | -0.017 | 0.61 | -0.001 | 0.87 | 0.01 | 0.22 |
| rs17787136 | *CRY2* | 0.014 | 0.75 | 0.054 | 0.22 | -0.0074 | 0.44 | -0.007 | 0.53 |
| rs11829762 | *CRY1* | -0.02 | 0.5 | -0.025 | 0.49 | 0.0036 | 0.59 | -0.014 | 0.15 |
| rs11113181 | *CRY1* | 0.029 | 0.31 | -0.015 | 0.62 | 0.006 | 0.35 | 0.012 | 0.13 |
| rs2304911 | *PER1* | -0.014 | 0.67 | 0.05 | 0.13 | 0.0072 | 0.31 | 0.006 | 0.47 |
| rs135764 | *CSNK1D* | -0.063 | 0.078 | 0.05 | 0.2 | 0.015 | 0.058 | 0.022 | **0.021** |

BMI: body mass index, SBP: systolic blood pressure, DBP: diastolic blood pressure, TG: triglycerides; Bold indicates *P*< 0.05. All of these traits were log-transformed in the regression analysis
